# Ketamine and Ceftriaxone-Induced Alterations in Glutamate Levels Do Not Impact the Specific Binding of Metabotropic Glutamate Receptor Subtype 5 Radioligand [^18^F]PSS232 in the Rat Brain

**DOI:** 10.3390/ph11030083

**Published:** 2018-08-29

**Authors:** Adrienne Müller Herde, Silvan D. Boss, Yingfang He, Roger Schibli, Linjing Mu, Simon M. Ametamey

**Affiliations:** 1 Center for Radiopharmaceutical Sciences of ETH, PSI, and USZ, Department of Chemistry and Applied Biosciences of ETH, 8093 Zurich, Switzerland; adrienne.herde@pharma.ethz.ch (A.M.H.); silvanboss@hotmail.com (S.D.B.); yingfang.he@pharma.ethz.ch (Y.H.); roger.schibli@pharma.ethz.ch (R.S.); linjing.mu@pharma.ethz.ch (L.M.); 2 Department of Nuclear Medicine, University Hospital Zurich, 8091 Zurich, Switzerland

**Keywords:** glutamate, metabotropic glutamate receptor subtype 5, [^18^F]PSS232, ketamine, ceftriaxone, positron emission tomography, allosteric modulator, MMPEP, ABP688

## Abstract

Several studies showed that [^11^C]ABP688 binding is altered following drug-induced perturbation of glutamate levels in brains of humans, non-human primates and rats. We evaluated whether the fluorinated derivative [^18^F]PSS232 can be used to assess metabotropic glutamate receptor 5 (mGluR5) availability in rats after pharmacological challenge with ketamine, known to increase glutamate, or ceftriaxone, known to decrease glutamate. In vitro autoradiography was performed on rat brain slices with [^18^F]PSS232 to prove direct competition of the drugs for mGluR5. One group of rats were challenged with a bolus injection of either vehicle, racemic ketamine, S-ketamine or ceftriaxone followed by positron emission tomography PET imaging with [^18^F]PSS232. The other group received an infusion of the drugs during the PET scan. Distribution volume ratios (DVRs) were calculated using a reference tissue model. In vitro autoradiography showed no direct competition of the drugs with [^18^F]PSS232 for the allosteric binding site of mGluR5. DVRs of [^18^F]PSS232 binding in vivo did not change in any brain region neither after bolus injection nor after infusion. We conclude that [^18^F]PSS232 has utility for measuring mGluR5 density or occupancy of the allosteric site in vivo, but it cannot be used to measure in vivo fluctuations of glutamate levels in the rat brain.

## 1. Introduction

Glutamate is the principle excitatory neurotransmitter in the nervous system, which elicits its action on ionotropic (*N*-methyl-d-aspartate receptor (NMDA), Kainate, α-amino-3-hydroxy-5-methyl-4-isoxazolepropionic acid receptor (AMPA)) and metabotropic (mGluR) receptors, especially in the mammalian brain [1]. Ionotropic receptors form ion channels and exhibit a fast relay. Metabotropic receptors are G protein-coupled, acting through a second messenger and produce stimuli that are more prolonged. Since glutamate is not degraded in the synaptic cleft, two major astrocytic transporters, the glutamate transporter-1 (GLT-1, EAAT2) and the glutamate aspartate transporter (GLAST, EAAT1), remove glutamate and provide the regulation to orchestrate receptor excitability [2]. Glutamate works not only as a point-to-point transmitter, but also through spill-over synaptic crosstalk between synapses in which summation of glutamate released from a neighboring synapse creates extrasynaptic signaling [3]. A disruption of these fine-tuning mechanisms can cause excitotoxicity, which is a driving force in the pathophysiology of various neuropsychological disorders. An important role of the glutamatergic system has been established in depression [4], schizophrenia [5], Parkinson’s disease [6], Alzheimer’s disease [7] and drug addiction [8]. Due to the involvement of the glutamatergic system in a large array of diseases, intense efforts have been made in recent years to visualize acute fluctuations in endogenous glutamate levels. Positron emission tomography (PET) using the highly selective allosteric antagonist of mGluR5, 3-(6-methyl-pyridin-2-ylethynyl)-cyclohex-2-enone-*O*-(11)C-methyl-oxime ([^11^C]ABP688) [9], has been used to measure in vivo receptor availability and fluctuations of endogenous glutamate. Several studies have shown that [^11^C]ABP688 binding is altered following drug-induced perturbation of glutamate levels in humans [10,11,12], baboons [13], rhesus monkeys [14] and rats [15]. In a pharmacological challenge study by Wyckhuys et al. [16], *N*-acetylcysteine (NAc), a compound which facilitates the activity of the cysteine–glutamate antiporter and indirectly increases extrasynaptic glutamate levels [17] did not affect the in vivo binding of [^11^C]ABP688 in the rat brain. In another pharmacological challenge study, Zimmer et al. [15] used the drug ceftriaxone, a potent GLT-1 activator [18], which induces a decrease in extracellular glutamate levels. Ceftriaxone selectively increases the expression of glial GLT-1 and protects neurons against ischemia via upregulation of GLT-1 following increased uptake of glutamate. This leads to diminished excitotoxicity of glutamate and protection of neurons against ischemia [19]. In their publication, Zimmer et al. reported an increase in [^11^C]ABP688 binding potential in the thalamic ventral anterior nucleus of the rat brain, however, the global binding potential (whole brain) was not changed under baseline and ceftriaxone challenge. No information was provided on any other rat brain compartment besides the thalamic ventral anterior nucleus.

We here present our study assessing the feasibility of using the novel ^18^F-labelled mGluR5 antagonist [^18^F]PSS232 [20] to measure in vivo fluctuation of endogenous glutamate levels in the rat brain by PET imaging. The results of this study should potentially shed more light on the utility of [^18^F]PSS232 for measuring mGluR5 availability after drug-induced perturbation of glutamate levels in the rat brain. We already reported on the kinetics of [^18^F]PSS232 in the rat brain and established the model-independent area-under-the-curve ratio method for robust quantification of PET results [21].

Given the contradictory results obtained in rats using [^11^C]ABP688, we applied two different pharmacological challenges of altering glutamate levels. We used (i) subanesthetic doses of ketamine, an NMDA receptor antagonist known to increase glutamate release [22,23] and (ii) the GLT-1 activator ceftriaxone, known to reduce glutamate levels. Furthermore, we compared the effects of racemic and S-ketamine on mGluR5 availability since a number of studies have suggested a two-fold higher potency for S-ketamine compared to racemic or R-ketamine [24,25,26]. Finally, in contrast to previous studies that used bolus injections to modulate glutamate release, we compared the effect of bolus injection and infusion of the three drugs.

## 2. Results

### 2.1. In Vitro Effects of Racemic Ketamine, S-Ketamine or Ceftriaxone on [^18^F]PSS232 Binding

**Figure 1:**
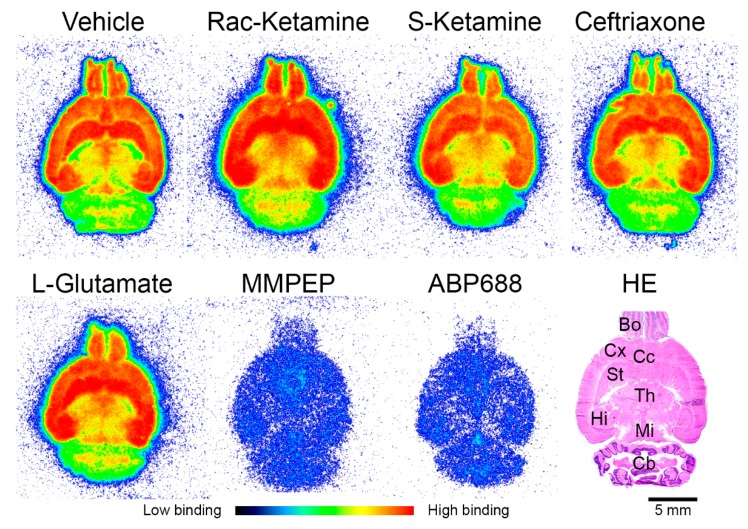
Representative in vitro autoradiograms of a rat brain incubated with 1 nM [^18^F]PSS232 solution supplemented with vehicle (0.9% NaCl), racemic ketamine, S-ketamine, ceftriaxone, L-glutamate, MMPEP ((2-[2-(3-methoxyphenyl)ethynyl]-6-methylpyridine hydrochloride) or ABP688 ((*Z*)-*N*-methoxy-3-[2-(6-methylpyridin-2-yl)ethynyl]-cyclohex-2-en-1-imine) (each 1 µM). [^18^F]PSS232 binding was strong to bulbus olfactorius (Bo), cortex (Cx), cortex cingulate (Cc), striatum (St) and hippocampus (Hi). Moderate binding was observed to thalamus (Th) and midbrain (Mi) and low binding to cerebellum (Cb). Color bar indicates low and high binding. Hematoxylin/eosin (HE) staining shows rat brain morphology. Scale bar: 5 mm.

To exclude direct binding of either racemic ketamine, S-ketamine or ceftriaxone to mGluR5, we performed in vitro autoradiography using rat brain slices. Figure 1 shows [^18^F]PSS232 binding to mGluR5-rich regions such as the striatum, hippocampus, cortex, cortex cingulate and bulbus olfactorius. The cerebellum with low mGluR5 density showed less [^18^F]PSS232 binding. This binding pattern was not altered when the brain slices were co-incubated with either racemic ketamine, S-ketamine or ceftriaxone compared to the vehicle. This result indicates that there is no direct interference of these pharmaceuticals with the binding site of [^18^F]PSS232 to mGluR5. In addition, after co-incubation of rat brain slices with L-glutamate, we found no competition between L-glutamate and [^18^F]PSS232 binding to mGluR5. As expected, the binding of [^18^F]PSS232 was abolished upon co-incubation with the two mGluR5 antagonists, MMPEP and ABP688.

### 2.2. In Vivo Effects of Racemic Ketamine, S-Ketamine or Ceftriaxone on [^18^F]PSS232 Binding

**Figure 2:**
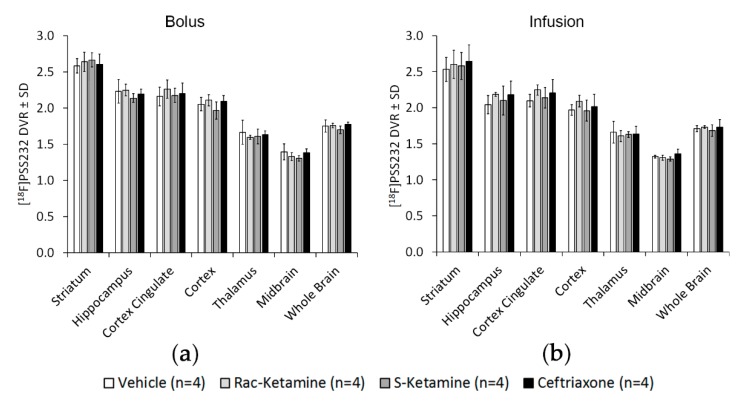
Influence of (**a**) bolus injection or (**b**) infusion of vehicle (0.9% NaCl), racemic ketamine, S-ketamine and ceftriaxone on [^18^F]PSS232 distribution volume ratios (DVRs) in different rat brain regions. Number of animals as depicted. SD, standard deviations.

**Figure 3:**
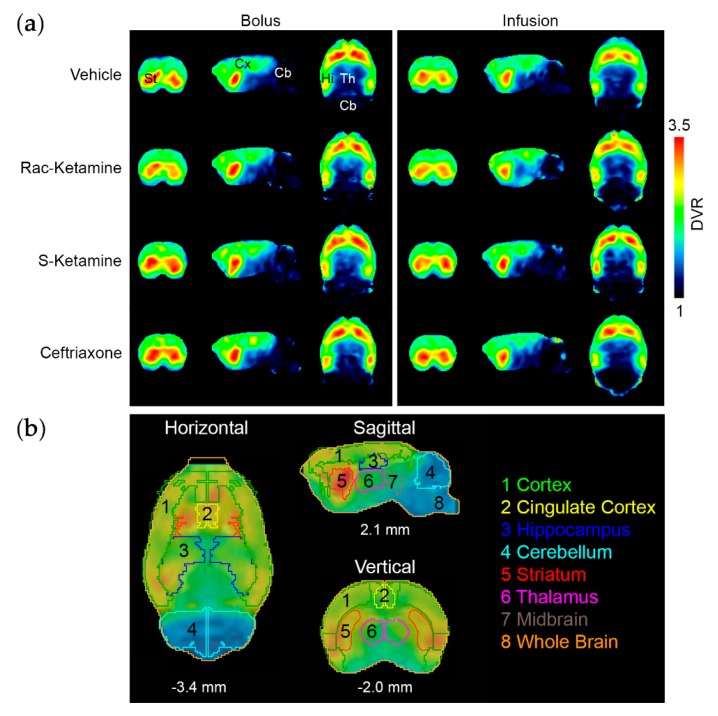
(**a**) illustration of averaged [^18^F]PSS232 brain uptake as mGluR5 distribution volume ratios (DVRs) after vehicle (0.9% NaCl; *n* = 4), racemic ketamine (*n* = 4), S-ketamine (*n* = 4) and ceftriaxone (*n* = 4) challenge. PET images were derived after bolus injection (**left**) or infusion (**right**) and presented in axial, sagittal and coronal planes. St, striatum; Cx, cortex; Cb, cerebellum; Hi, hippocampus; Th, thalamus; (**b**) definition of brain regions-of-interest on PET images in horizontal (coronal), sagittal and vertical (axial) sections. Locations in mm are distance to the Bregma. The quantitative regions-of-interest analysis is shown in Figure 2.

To measure ketamine- or ceftriaxone-induced changes in mGluR5 availability as an index of glutamate release, we used [^18^F]PSS232 PET imaging applying a bolus injection protocol. In Figure 2a, the mGluR5 distribution volume ratios (DVRs) for selected brain regions are depicted. For all three drugs, the highest mGluR5 DRVs were found in the striatum (2.6 ± 0.03) followed by the hippocampus (2.2 ± 0.04), cortex cingulate (2.2 ± 0.04) and cortex (2.1 ± 0.06). Global mGluR5 DVR for the three drugs, racemic ketamine, S-ketamine and ceftriaxone, did not change and was similar for the whole brain (1.7 ± 0.03). We found no significant between-group differences after bolus injections. Based on human studies with [^11^C]ABP688 that reported reduced mGluR5 availability after infusion of ketamine [10,12], we adjusted our study protocol and infused ketamine or ceftriaxone after an initial bolus injection. Figure 2b shows the DVRs for different brain regions and the whole brain after the different challenges, which, however, did not significantly differ from each other. Similar to the bolus protocol, mGluR5 DVRs were highest in the striatum (2.6 ± 0.02) followed by the hippocampus (2.1 ± 0.08), cortex cingulate (2.2 ± 0.05) and cortex (2.0 ± 0.05). Global mGluR5 DVR (1.7 ± 0.01) was not affected by the different challenges and intergroup differences were also not significant. Figure 3a shows parametric DVR images of the rat brain using bolus and infusion protocols. There were no differences in [^18^F]PSS232 binding in either ketamine- or ceftriaxone-induced glutamate fluctuations or injection protocols. Figure 3b shows the brain regions-of-interest defined to calculate the DVRs.

**Figure 4:**
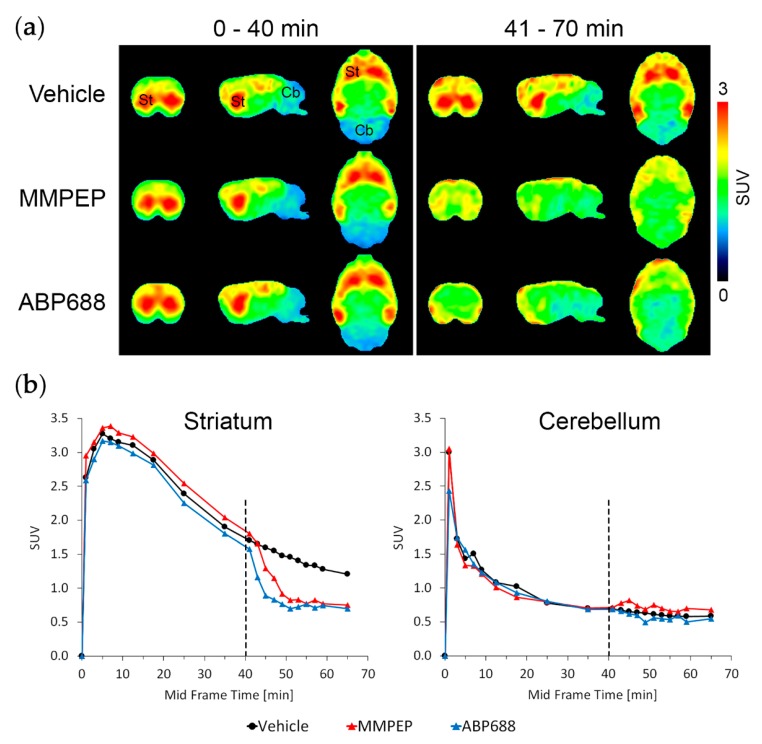
In vivo displacement study of [^18^F]PSS232 with either vehicle (*n* = 1; 1:1 PEG200/aqua ad inject), 1 mg/kg MMPEP (*n* = 1) or 1 mg/kg ABP688 (*n* = 1) at 40 min post radiotracer injection. (**a**) [^18^F]PSS232 PET images of rat brains averaged from 0–40 and 41–70 min after radiotracer injection. PET images are presented in axial, sagittal and coronal planes. St, striatum; Cb, cerebellum; (**b**) time–activity curves for [^18^F]PSS232 in the mGluR5-rich striatum and mGluR5-low cerebellum. Dashed line at 40 min represents the time point of injection of the vehicle, MMPEP ((2-[2-(3-methoxyphenyl)ethynyl]-6-methylpyridine hydrochloride) or ABP688 ((*Z*)-*N*-methoxy-3-[2-(6-methylpyridin-2-yl)ethynyl]-cyclohex-2-en-1-imine). SUV, standardized uptake values.

In order to demonstrate reversibility of binding of [^18^F]PSS232 in vivo, displacement studies, using MMPEP and ABP688, which were applied 40 min after [^18^F]PSS232 injection, were performed. The results showed a rapid wash-out of radioactivity from mGluR5-rich brain regions (Figure 4a). Striatal radioactivity values decreased shortly after MMPEP or ABP688 administration to the cerebellar level and remained low (Figure 4b).

## 3. Discussion

The goal of this study was to investigate whether [^18^F]PSS232 can be used to image glutamate fluctuations as was, to some extent, reported for [^11^C]ABP688. [^18^F]PSS232 is a fluorinated derivative of [^11^C]ABP688, which binds to the same site in the transmembrane domain of mGluR5 as [^11^C]ABP688. In our experimental setup, we induced glutamate increase by injection of ketamine and glutamate decrease by activating GLT-1 with ceftriaxone. Several publications report significant changes in endogenous extraneuronal glutamate levels by ketamine and ceftriaxone [12,15,22]. In our study, we used ketamine dosages (25 mg/kg bolus, 0.6 mg/kg/h infusion) known to be high enough to increase extraneuronal glutamate levels, as measured in vivo with microdialysis [12,22]. Ketamine increases glutamate directly by influencing mGluR5 functional status and through the antagonism of NMDA receptors. Furthermore, mGluR5 and NMDA receptors functionally interact and mutually potentiate their responses [27,28]. The dosage of the bolus-injected ceftriaxone (200 mg/kg) used in this study was also reported to be high enough to decrease glutamate levels, at least in the thalamic ventral anterior nucleus of the rat brain by micro-PET and microdialysis [15]. Changes in glutamate levels after infusion of ceftriaxone have not been reported yet. Although GLT-1 and mGluR5 are expressed in almost all brain regions, splice variants or differential expression of GLT-1 may explain the distinct activation by ceftriaxone. Our experiments demonstrated that the mGluR5-specific radiotracer, [^18^F]PSS232, was not able to detect changes in endogenous glutamate levels induced by ketamine or ceftriaxone in the rat brain in vivo. Our in vitro autoradiography results showed that neither ketamine nor ceftriaxone or glutamate itself competed with [^18^F]PSS232 for binding to mGluR5. However, the allosteric antagonists MMPEP and unlabeled ABP688 reduced [^18^F]PSS232 binding in vitro and in vivo. The in vivo displacement studies confirmed that [^18^F]PSS232 binds to the allosteric site of mGluR5. Our in vivo findings indicate that the altered levels of endogenous glutamate did not alter the capacity of [^18^F]PSS232 to bind to mGluR5 in the rat brain. We hypothesized that changes in endogenous glutamate would lead to conformational changes of mGluR5 resulting in an increased or decreased affinity of [^18^F]PSS232. However, our results indicated no affinity shift of [^18^F]PSS232 to mGluR5 in vivo. The binding of [^18^F]PSS232 to the allosteric site instead of the orthosteric binding site of mGluR5 might be dependent on the tertiary and quaternary receptor conformations [29]. Oligomeric and heteromeric forms of mGluR5 might influence the availability of the allosteric site [30,31]. Furthermore, changes of in vivo glutamate levels might alter mGluR5 conformational states, which were, however, not supported by our findings. On the other hand, affinity shift in receptor–radioligand interactions was described for dopamine D2 receptors, where amphetamine challenge altered the binding of a D2 PET radioligand [32,33].

To the best of our knowledge, this is the first study reporting on the potential of [^18^F]PSS232 to image glutamate fluctuations. In contrast to our results, a recent study in rats indicated that [^11^C]ABP688 was able to visualize acute fluctuation in endogenous glutamate levels after challenge with ceftriaxone [15]. Ceftriaxone administration increased [^11^C]ABP688 binding potential in the thalamic ventral anterior nucleus bilaterally, but not in the frontal cortex. The authors have, so far, no explanation why this thalamic region was the first to be affected by ceftriaxone and suggested further investigations. We investigated the thalamic area but found no changes in mGluR5 DVRs after vehicle, ceftriaxone or ketamine administration. To date, no publication is available that has reported on ketamine-induced glutamate fluctuations and consequent alterations in mGluR5 availability using PET imaging in the rat brain. Most of the ketamine challenges were performed in humans demonstrating a rapid and large (20%) reduction in [^11^C]ABP688 binding after ketamine infusion in healthy humans [10]. The ketamine-induced decrease in [^11^C]ABP688 binding was explained by a reduction in mGluR5 availability due to internalization. This internalization might be in response to glutamate release or a glutamate-induced conformational change in the receptor that reduces the likelihood of radioligand binding at the allosteric site. Other approaches to increase endogenous glutamate have been made with NAc in rats, rhesus monkeys and baboons. In baboons, NAc decreased [^11^C]ABP688 binding potential, which may be the result of an affinity shift in the binding to the allosteric site [13]. In rats [16] and rhesus monkeys [14], the binding of [^11^C]ABP688 was not affected after NAc challenge. In the rat study, the authors applied the NMDA receptor antagonist MK-801, which has a similar action as ketamine, and found no changes in [^11^C]ABP688 binding [16]. Although the authors used a different pharmacological approach, their results are in line with our findings. Discrepancies among the different studies could be species differences and variations in the methodology.

## 4. Materials and Methods

### 4.1. Animals

All procedures were performed according the Guide to the Care and Use of Experimental Animals of the Swiss legislation on animal welfare. All procedures fulfilled the ARRIVE guidelines on reporting animal experiments and complied with the commonly-accepted ‘3Rs’. The protocols for pharmacological administration and PET imaging were approved by the Veterinary Office of the Canton Zurich, Switzerland (permit number: ZH017/2015). Male Wistar (Crl:WI) rats were purchased from Charles River (Sulzfeld, Germany) and their body weights were between 350 g and 420 g at the time of the experiments. Rats were kept in a room with controlled temperature (21 °C) under a 12-h light/12-h dark cycle, with ad libitum access to food and water.

### 4.2. Radiosynthesis and Pharmaceuticals

**Scheme 1:**
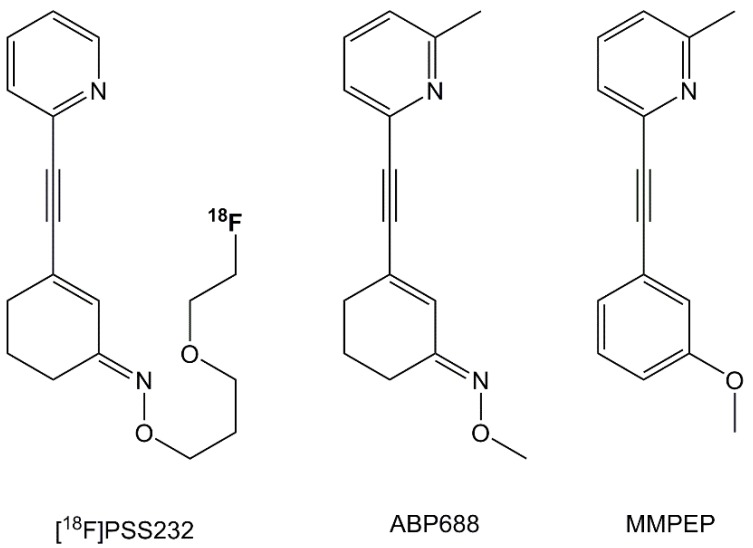
Chemical structures of the mGluR5 radiotracer [^18^F]PSS232 and mGluR5 antagonists ABP688 ((*Z*)-*N*-methoxy-3-[2-(6-methylpyridin-2-yl)ethynyl]-cyclohex-2-en-1-imine) and MMPEP ((2-[2-(3-methoxyphenyl)ethynyl]-6-methylpyridine hydrochloride).

The radiosynthesis of [^18^F]PSS232 was conducted as described previously [20]. The mean molar radioactivity at end of synthesis was 95.3 ± 6.2 GBq/µmol. Racemic ketamine hydrochloride was obtained from LGC (MM0144.00) (Teddington, UK), S-ketamine hydrochloride from Cayman Chemical (CAY-9001961) (Ann Arbor, MI, USA), ceftriaxone from Sigma Aldrich (C5793) (St. Louis, MO, USA), L-glutamate from Sigma Aldrich (128430), and 0.9% NaCl from B.Braun (Melsungen, Germany). MMPEP (2-[2-(3-methoxyphenyl)ethynyl]-6-methylpyridine hydrochloride) and ABP688 ((*Z*)-*N*-methoxy-3-[2-(6-methylpyridin-2-yl)ethynyl]-cyclohex-2-en-1-imine) were produced in-house. For in vivo administration, both forms of ketamine were dissolved in aqua ad inject and ceftriaxone in 0.9% NaCl. MMPEP and ABP688 were dissolved in 1:1 PEG200/aqua ad inject. Isoflurane (Isocare, Animalcare, York, UK) was used as an anesthetic agent. Scheme 1 shows the chemical structures of [^18^F]PSS232, ABP688 and MMPEP.

### 4.3. In Vitro Autoradiography

Coronal Wistar rat brain sections (10 μm) were cut on a cryostat (CryoStar NX50, Thermo Scientific, Waltham, MA, USA) and mounted on glass slides (Superfrost Plus, Thermo Scientific). Consecutive sections were thawed on ice for 10 min before pre-incubation in HEPES/BSA-buffer (4-(2-hydroxyethyl)-1-piperazineethanesulfonic acid/bovine serum albumin) (30 mM HEPES, 1.2 mM MgCl_2_, 110 mM NaCl, 2.5 mM CaCl_2_, 5 mM KCl, pH 7.4, 0.1% BSA) at 4 °C for another 10 min. Excess solution was carefully removed and tissue slices were incubated with 1 nM [^18^F]PSS232 supplemented with either vehicle (0.9% NaCl), 1 µM racemic ketamine, 1 µM S-ketamine, 1 µM ceftriaxone, 1 µM L-glutamate, 1 µM mGluR5 antagonist MMPEP or 1 µM mGluR5 antagonist ABP688. After incubation for 40 min at room temperature in a wet chamber, the slices were washed in ice cold HEPES/BSA-buffer for 5 min, twice in HEPES-buffer for 2 min each and finally dipped twice in distilled water. Dried slices were exposed to a phosphor imager plate (BAS-MS 2025, Fuji, Dielsdorf, Switzerland) for 15 min and the plate was scanned in a BAS-5000 reader (Fuji). A consecutive section was stained with hematoxylin (Gill No. 1, Sigma) and eosin (Eosin Y, Sigma) (HE) and digitalized by a slide scanner (Pannoramic 250, Sysmex, Horgen, Switzerland) to obtain brain morphology.

### 4.4. In Vivo PET Imaging

In vivo positron emission tomography/computed tomography (PET/CT) scans were performed with a calibrated Super Argus scanner (Sedecal, Madrid, Spain). Rats were anesthetized with isoflurane (2% isoflurane at 0.4 L/min oxygen:air (1:1) flow). Respiratory rate and temperature were controlled during the whole scan period (SA Instruments, Inc., Stony Brook, NY, USA). Rats were placed in a prone position and the brain was positioned in the center of the field of view. All intravenous (i.v.) injections were conducted via tail vein.

### 4.5. Bolus and Infusion Protocol

**Figure 5:**
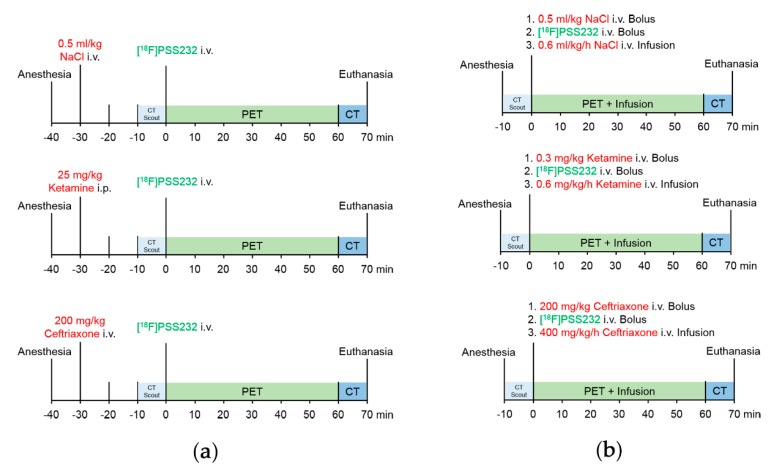
Glutamate challenge study design. (**a**) bolus protocol: anesthetized rats were injected either with 0.5 mL/kg vehicle intravenously (0.9% NaCl i.v.), 25 mg/kg racemic or S-ketamine intraperitoneally (i.p.) or 200 mg/kg ceftriaxone i.v. at 20 min before a short computed tomography (CT scout) or 30 min before injection of [^18^F]PSS232 i.v., respectively. Positron emission tomography (PET) scans lasted for 60 min followed by CT scan; (**b**) infusion protocol: anesthetized rats received a short CT scout and were injected i.v. with either 0.5 mL/kg vehicle (0.9% NaCl), 0.3 mg/kg racemic or S-ketamine or 200 mg/kg ceftriaxone. Administration of [^18^F]PSS232 i.v. preceded the infusion of either 0.6 mL/kg/h vehicle (0.9% NaCl), 0.6 mg/kg/h racemic or S-ketamine or 400 mg/kg/h ceftriaxone. PET acquisition and infusion lasted for 60 min followed by a CT scan.

For the bolus injection protocol (Figure 5a), rats were injected with either 0.5 mL/kg vehicle i.v. (0.9% NaCl; *n* = 4), 25 mg/kg racemic ketamine (*n* = 4) intraperitoneal (i.p.), 25 mg/kg S-ketamine (*n* = 4) i.p. or 200 mg/kg ceftriaxone (*n* = 4) i.v. at 30 min before i.v. injection of [^18^F]PSS232 (28.0 ± 2.1 MBq, 3.3 ± 2.0 nmol/kg, 87.8 ± 9.8 GBq/µmol). A short CT scout preceded the PET acquisition that lasted for 60 min and was followed by a CT scan to obtain anatomical orientation.

For the infusion protocol (Figure 5b), rats received a short CT scout and were injected i.v. with either 0.5 mL/kg vehicle (0.9% NaCl; *n* = 4), 0.3 mg/kg racemic ketamine (*n* = 4), 0.3 mg/kg S-ketamine (*n* = 4) or 200 mg/kg ceftriaxone (*n* = 4). Administration of [^18^F]PSS232 i.v. (28.5 ± 3.2 MBq, 3.6 ± 2.4 nmol/kg, 95.2 ± 7.8 GBq/µmol) preceded the infusion of either 0.6 mL/kg/h vehicle (0.9% NaCl), 0.6 mg/kg/h racemic ketamine, 0.6 mg/kg/h S-ketamine or 400 mg/kg/h ceftriaxone. PET acquisition lasted for 60 min followed by a CT scan. At the end of the scans, rats were euthanized.

### 4.6. In Vivo Displacement Study

For displacement studies of [^18^F]PSS232 from mGluR5 binding sites either 1 mL/kg vehicle (1:1 PEG200/aqua ad inject), 1 mg/kg MMPEP or 1 mg/kg ABP688 was i.v. injected at 40 min after [^18^F]PSS232 i.v. injection (30.1 ± 3.2 MBq, 3.0 ± 1.9 nmol/kg, 103.6 ± 4.8 GBq/µmol). We chose this relatively late time point to guarantee radiotracer equilibration in the brain. The total duration of the PET scan was 70 min followed by a CT scan.

### 4.7. Image Data Reconstruction, Analysis and Calculation of Distribution Volume Ratios (DVRs) as Well as Standardized Uptake Values (SUVs)

PET data were reconstructed in user-defined time frames with a voxel size of 0.3875 × 0.3875 × 0.775 mm^3^ by two-dimensional-ordered subsets expectation maximization (2D-OSEM). Random and single but no attenuation correction was applied. Image files were analyzed with PMOD 3.8 software (PMOD Technologies Ltd., Zurich, Switzerland). For calculation of DVRs in regions-of-interest, specific brain regions were defined on the bases of the rat MRI T2 template (provided with PMOD software) (Figure 3b). Given that hardly any specific binding was found in rat cerebellum, this brain region was used as a reference region. DVRs were calculated from areas-under-the-curve as described recently [21]. Individual DVR images were calculated voxel-wise with the PXMod module of PMOD by means of the implemented reference Logan model and the cerebellum as reference region. Individual DVR images of the 4 animals per group were combined and averaged with the View module of PMOD.

For calculation of the time–activity curves for striatum and cerebellum, both regions were defined on the rat MRI T2 template. The tissue radioactivity values of both brain regions were decay-corrected and normalized to the injected radioactivity and body weight resulting in SUVs.

### 4.8. Statistical Analysis

Differences in mean values were evaluated by one-way analysis of variance (ANOVA) tests, followed by corrections for multiple testing (Bonferroni) using GraphPad Prism 6.0 software (GraphPad, La Jolla, CA, USA). A *p*-value < 0.05 was considered significant.

## 5. Conclusions

The present study verified that [^18^F]PSS232 binds to delineated structures in the rat brain; however, [^18^F]PSS232 does not seem to be a valuable tool for the in vivo assessment of acute endogenous glutamate fluctuations. Neither increased glutamate levels after ketamine challenge with changes in the functional status of mGluR5 through inhibition of the NMDA receptor nor decreased glutamate levels after challenge with GLT-1 activator ceftriaxone had any effect on the binding of [^18^F]PSS232 to mGluR5. Whether [^18^F]PSS232 will show similar effects in humans as demonstrated for [^11^C]ABP688 remains to be validated in future clinical studies.
